# Biophilic Design and Restorative Effects: A Neuropsychological Study of Healthy Indoor Workspaces in Urban Contexts

**DOI:** 10.3390/ijerph22101571

**Published:** 2025-10-15

**Authors:** ChoHye Youn, Minji Kang, Juyoung Lee

**Affiliations:** 1 Department of Landscape Architecture, Hankyong National University, Anseong 17579, Republic of Korea; fot5577@naver.com; 2 Division of Forest Human Service Research, National Institute of Forest Science, Seoul 02455, Republic of Korea

**Keywords:** urbanicity, built environment, healthy place, green element, restorativeness

## Abstract

The rapid increases in urbanization and time spent in built indoor environments have sparked significant concerns about their impact on human health and well-being. People who spend long hours in enclosed and highly artificial settings, such as hospital workers, are especially vulnerable to environmental stressors. This study examined whether applying biophilic (i.e., human tendency to connect with nature and other lifeforms) spatial design within hospital spaces could provide restorative benefits for nurses working in high-stress environments. Twenty-one nurses participated in this study, staying under two different conditions for 10 min: a control room with plain white walls and a biophilic space where the walls were entirely covered with vegetation. During the sessions, functional near-infrared spectroscopy was used to measure hemodynamic responses in the dorsolateral prefrontal cortex (DLPFC). Additionally, standardized self-report questionnaires were used to analyze the level of perceived restorativeness, affective states, mood, and anxiety. Results showed that exposure to the biophilic space significantly reduced oxyhemoglobin (oxy-Hb) concentrations in the DLPFC, reflecting relief from cognitive–emotional overload and enhanced neural stabilization. Psychological measures further indicated decreases in fatigue, depression, and anxiety, alongside increases in vigor, attentiveness, and perceived restorativeness in biophilic space. These findings demonstrate that even brief exposure to a biophilically designed indoor environment can produce neuropsychologically restorative effects, suggesting biophilic interventions as sustainable, effective strategies for healthier workplaces and urban environments.

## 1. Introduction

The relationship between the environment and human health is a critical issue when considering urban sustainability. As urban populations continue to grow, this issue has drawn considerable attention from the international community. The United Nations projected that nearly 70% of the global population will reside in urban areas by 2050 and has designated “Good Health and Well-Being” as a central objective in its Sustainable Development Goals [1]. This emphasizes that the creation of healthy urban environments represents critical social imperatives that directly influence human quality of life.

Urbanization has accelerated the spread of built environments, which are structures designed and constructed by humans [2]. Artificial environments are characterized by rigidity and a lack of vitality, which exert negative influences on humans’ physical and mental health and on broader social and environmental dimensions, thereby increasing societal burdens [3]. Rapid urbanization and the spread of artificial environments correlate with decreased quality of life and higher risk of chronic health problems [4,5].

In contrast, natural environments—rich in vitality and diversity—play a crucial role in fostering human recovery and well-being. Extensive evidence indicates that exposure to nature contributes to stress reduction, cognitive restoration, and emotional stability [6,7,8]. These outcomes are grounded in the Biophilia Hypothesis, which, from an evolutionary psychology perspective, posits that humans possess an innate tendency to seek affiliation with nature as a foundation for survival and psychological balance [9]. This perspective provides the theoretical basis for biophilic design, which integrates natural elements into built environments to promote health and resilience [10]. Recent studies emphasize the importance of applying biophilic principles across diverse spatial scales and ensuring equitable access to restorative green spaces in urban settings [11,12,13,14].

Modern life has become increasingly confined to indoor settings where individuals are exposed to monotonous physical environments and isolated from natural elements. These conditions constrain sensory diversity and contribute to heightened psychological stress as well as impaired cognitive functioning [15]. In particular, occupational groups that spend extended hours in enclosed indoor environments are likely to experience exacerbated effects. For instance, healthcare workers in hospitals face the spatial restrictions of closed artificial settings and excessive workloads, which can accumulate significant psychological and physical strain [16,17]. This indicates that individuals may be substantially influenced psychologically and physically by the physical environment of a workplace.

Strong evidence regarding the relationship between the natural environment and human health has raised attention to nature-based solutions as strategies for improving urban public health. The natural elements in the workplace can positively affect employees’ mental health and cognitive performance [18]. Exposure to natural environments is associated with stabilized autonomic nervous activity, reduced negative affect, and enhanced positive emotions compared to urban settings [19].

It has also been reported that nature-based stimuli significantly lower cortisol levels and facilitate recovery responses in individuals undergoing rehabilitation [20]. Consequently, urban planning and landscape architecture have increasingly emphasized the integration of natural elements as an effective strategy to enhance human health and well-being, with the significance of restorative environments gaining growing attention [21].

With the development of environmental neuroscience, recent research has increasingly sought to analyze the effects of physical environmental stimuli on brain function [22]. Studies using functional magnetic resonance imaging (fMRI) have demonstrated that urban environments facilitate stress sensitivity by increasing amygdala activation, whereas exposure to natural environments reduces amygdala activity and induces psychological stability [23,24,25]. Functional near-infrared spectroscopy (fNIRS), as a non-invasive technique, has played a crucial role in measuring prefrontal cortex (PFC) hemodynamics in real time. It has also been used to elucidate PFC responses when exposed to natural environments in connection with cognition and emotion regulation.

Previous studies have consistently shown that exposure to natural environments can induce measurable benefits for human health and cognition. For instance, indoor nature exposure has been found to enhance resilience and cognitive functioning [26], while bilateral prefrontal hemodynamic responses were reduced, reflecting relief from excessive cortical load [27]. In line with these findings, decreased activation in the ventral and orbitofrontal cortices has also been observed, indicative of stress alleviation and functional stabilization [28]. Similarly, viewing natural images was shown to decrease hemodynamic responses in the right orbitofrontal cortex (OFC), reflecting reduced emotional strain and increased psychological stability [29]. Other studies have reported that gardens, compared to urban landscapes, led to bilateral prefrontal deactivation, a marker of relaxation and negative affect reduction [30], and that forest imagery significantly decreased right prefrontal activation, accompanied by enhanced psychological comfort [31]. Likewise, participants exposed to indoor plants exhibited reduced prefrontal responses, signifying functional efficiency rather than impaired activity [32].

The PFC can be divided into distinct areas with specialized functions [33]. Within these, the dorsolateral prefrontal cortex (DLPFC) appears to be particularly reactive to emotional responses linked to environmental factors [34,35]. It has been reported that DLPFC activation is often associated with negative emotions, and natural stimuli stabilize DLPFC responses by promoting emotional regulation and recovery.

fNIRS-based studies have shown that natural stimuli modulate PFC function to promote psychological recovery [26,27,28,29,30,31,32,36,37,38]. The integrative approach linking neural and psychological responses highlights the close relationship between the physical environment and mental health. It also provides new perspectives for creating healthier spaces in built environments. However, neurophysiological studies targeting populations exposed to highly artificial and confined indoor environments, such as hospital workers, remain scarce.

While studies have confirmed the stress-reducing benefits of nature exposure, little is known about its specific neural effects in such specialized contexts. In particular, there is a lack of research examining the function of the DLPFC in naturalized settings, a key hub for cognitive control, environmental stimulus processing, and higher-order emotional regulation [39,40,41,42]. Therefore, this study focused on investigating neuropsychological responses to the different types of physical environmental attributes that contribute to the concept of a healthy workplace. Specifically, it compared cognitive and psychological responses between artificially constructed indoor environments and naturalized settings, thereby providing evidence and insights into how biophilic design may influence restorativeness in the workplace context.

## 2. Materials and Methods

### 2.1. Participants

To evaluate the influences of two different types of indoor settings on cognitive characteristics in hospital spaces, prefrontal cortical reactivity and psychological responses were measured. The participants were 21 nurses working in a national hospital located in Cheon-An City, South Korea (mean age: 35.0 ± 7.9 years). The appropriate sample size was determined through a power test and by referencing previous studies with similar experimental designs. In previous neurophysiological studies utilizing fNIRS to investigate environmental stimuli, sample sizes typically ranged from 10 to 30 participants [23,26,27,29,30,31,32]. A within-subject repeated-measures design was adopted to minimize inter-individual variability and enhance statistical reliability. Adults without a history of neuropsychiatric or cardiovascular disorders were recruited through an internal hospital notice posted at the facility where the experiment took place, which included a brief description of the study’s purpose and procedures. Nurses who expressed interest contacted the principal investigator directly and provided written informed consent prior to participation. All participants completed experimental conditions and received a small token of appreciation upon finishing the study.

The study protocol was reviewed and approved by the Institutional Review Board of the Joint Institutional Bioethics Committee (P01-202110-12-003), and all procedures adhered to the principles of the Declaration of Helsinki.

### 2.2. Research Settings

**Figure 1:**
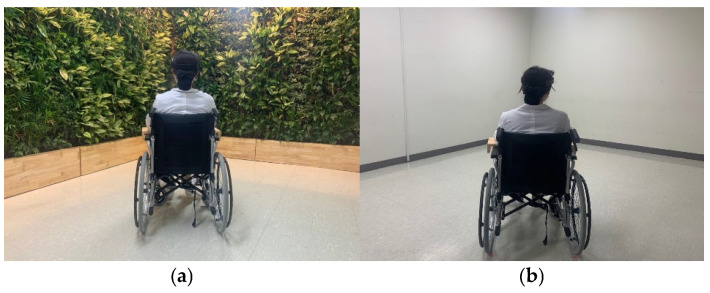
Experimental settings. (**a**) Biophilic space with walls entirely covered with vegetation. (**b**) Control space with plain white cement walls.

Two indoor spaces in a hospital were prepared. One was a control setting with plain white wall, and the other was a naturalized space with biophilic design where the walls were entirely covered with vegetation, providing a total green area of 21 m^2^ (Figure 1). The design was intentionally simplified to ensure a clearer interpretation and stronger internal validity of the results. In the biophilic space, 10 species of foliage plants were cultivated in individual pots and arranged to uniformly cover the wall surface, allowing the space to be visually perceived as a continuous biophilic area (Table 1). The control condition was established in a monotonous white wall with the same flooring material as the biophilic space. All experimental settings were cleared of all furniture and decorative elements. To minimize the influence of external environmental variables, experiments were conducted under controlled conditions. Temperature, humidity, and illuminance were maintained at 25.9 ± 0.6 °C, 61.5 ± 4.3%, and 373.0 ± 61.1 lx, respectively, in both the biophilic space and the control conditions.

### 2.3. Measurement

#### 2.3.1. Blood Flow Monitoring in the Prefrontal Cortex

To examine the differences in cognitive performance across the two contrasting spatial conditions, we assessed prefrontal cortical hemodynamics. Functional near-infrared spectroscopy (fNIRS) was utilized because this method allows for the region-specific evaluation of prefrontal cortical activity. fNIRS is a noninvasive technique that utilizes near-infrared light within the 650–1000 nm wavelength range to measure relative changes in oxyhemoglobin (oxy-Hb) and deoxyhemoglobin (deoxy-Hb) concentrations within biological tissue [43]. Near-infrared spectroscopy (NIRS) enables real-time monitoring of cerebral oxygen metabolism and hemodynamic responses, making it a valuable tool for assessing functional activity in regions involved in cognitive and emotional regulation, such as the PFC [44,45,46].

In this study, a portable NIRS device (NIRSIT Lite, OBELAB Inc., Seoul, Korea) was employed, which allowed stable data acquisition, even in environments permitting relatively free movement. The device is lightweight and designed for easy attachment and detachment, enabling its application not only in indoor experiments but also in dynamic settings [47].

Given the study’s objective, the analyses focused on the DLPFC. The DLPFC is a critical hub for higher-order cognitive functions, including attentional control and working memory, and is particularly sensitive to environmental stimuli, thereby serving as an appropriate target region for evaluating cognitive restoration and emotional responses.

#### 2.3.2. Quantitative Measurement of Psychological States

The Perceived Restorativeness Scale (PRS) was applied to evaluate the influences of biophilic design on restorativeness, a self-report questionnaire designed to measure the restorative qualities of specific environments. It consists of 16 items across four subdomains: being away, fascination, coherence, and compatibility. Higher scores indicate greater restorative potential for the environment. In the present study, the reliability analysis yielded a Cronbach’s α of 0.95, indicating excellent internal consistency.

The Zuckerman Inventory of Personal Reaction Scale (ZIPERS) was used to measure stress responses to the two types of indoor space. The ZIPERS is a 12-item measure comprising five subscales: positive affect, attentiveness, fear, anger, and sadness. Higher scores reflect stronger emotional responses in each respective domain. In this study, the Cronbach’s α was 0.70, representing an acceptable level of reliability.

A specific space can change the mood states of individuals experiencing mental fatigue and stress. The Profile of Mood States-Brief (POMS) is a 30-item questionnaire that assesses six mood dimensions: tension–anxiety, anger–hostility, depression, fatigue, confusion, and vigor. To minimize redundancy with other scales and reduce participant burden, only three subscales—vigor, fatigue, and depression—were used in this study. Although the overall Cronbach’s α of the full scale was 0.59, reflecting heterogeneity among factors, the reliability coefficients of the selected subscales were acceptable (fatigue = 0.77, vigor = 0.94, depression = 0.73). Therefore, subscale scores were employed in subsequent analyses.

Excessive workload and fatigue are commonly associated with heightened psychological anxiety, which can be modulated by the characteristics of the physical environment. The State-Trait Anxiety Inventory (STAI) measures state anxiety, with higher scores indicating greater levels of anxiety. This scale is particularly suited to capturing immediate emotional responses to specific environments. In the present study, Cronbach’s α was 0.92, demonstrating excellent internal consistency.

### 2.4. Procedure

**Figure 2:**
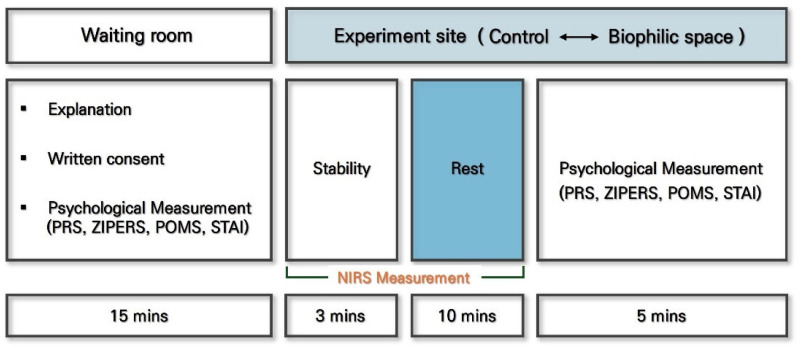
Experimental procedure and timeline under the control and biophilic conditions.

First, the participants were fully informed of the study’s purpose and procedures and provided written informed consent before participation. For measuring cerebral hemodynamics, NIRS probes were attached to the prefrontal region, positioned approximately 10–20 mm above both eyebrows while participants remained seated. Subsequently, the participants were moved to either the experimental or the control condition. To minimize physiological responses caused by body movement, all transfers and experimental procedures were conducted with participants seated in wheelchairs (Figure 2).

Each condition involved 10 min of exposure to the assigned environment (biophilic space vs. control), corresponding to the typical break duration in medical settings. During this period, participants remained seated and rested with their eyes open. The 10-min timeframe was determined based on empirical and practical considerations. Previous studies have demonstrated that short-term breaks can alleviate job stress among hospital nurses through psychological detachment [48], and that exposure to natural environments for at least 10 min can yield measurable improvements in mental health and cognitive functioning [19,49]. Also, in Korea, the minimum duration of a single rest break in workplace is generally at least 10 min. Therefore, a 10-min exposure period was adopted in this study to reflect a realistic and evidence-based rest duration for evaluating the short-term restorative effects of biophilic environments in workplace settings. To reduce residual physiological effects between conditions, a 3-min stabilization period was implemented before switching environments. After each session, the participants completed self-report questionnaires to assess their psychological responses to exposure to different conditions. The order of conditions was randomized to eliminate potential order effects, and all participants completed one of the measurements in both the biophilic space and the control conditions.

### 2.5. Data Analysis

This study was conducted with a total of 21 healthcare professionals, and raw data collected using the NIRSIT Quest program were preprocessed to examine the effects of biophilic space exposure on the DLPFC.

Raw intensity signals were first inspected, and invalid segments (≤5 consecutive samples) were interpolated, whereas channels with >5 consecutive invalid values were rejected. Channels were further excluded if their median intensity was <30 [50], the coefficient of variation exceeded 15% [51], or >5% of the time series consisted of identical consecutive values, indicating saturation. The remaining signals were converted to optical density [50] and corrected for motion artifacts using the Temporal Derivative Distribution Repair method [52].

Subsequently, data were transformed into oxy- and deoxyhemoglobin concentrations using the modified Beer–Lambert Law [53,54], with differential pathlength factors (780 nm = 6, 850 nm = 5.2) and extinction coefficients derived from Zhao et al. [55]. To reduce superficial physiological noise, short-channel regression was applied using channels with a source–detector distance ≤ 8 mm [56]. Finally, band-pass filtering (0.005–0.1 Hz) with a discrete cosine transform was performed [50]. Channels showing an extreme negative correlation between HbO and HbR (r ≤ –0.9) were rejected [57]. Missing data were padded with the mean of the remaining channels. This preprocessing pipeline ensured a reliable estimation of dorsolateral prefrontal hemodynamics.

For the DLPFC analysis, channels were extracted from NIRSIT Cluster 1, specifically the right channels (CH3, CH5, and CH6) and the left channels (CH9, CH11, and CH12). Changes in oxyhemoglobin (oxy-Hb) concentrations from these channels were calculated as both 10-min averages and 2-min interval averages. Following preprocessing, statistical analyses were performed using SPSS Statistics 29.0 (IBM, USA). Paired *t*-tests were applied for the analysis of DLPFC hemodynamics, while Wilcoxon signed-rank tests were used for the psychological data. All analyses were conducted within-subject design, as all participants completed both experimental conditions. For the paired *t*-test, differences between the biophilic and control conditions were calculated for each participant, and the *t*-value was obtained as:

$$t=\frac{\bar{d}}{s_{d}/\sqrt{n}},\;\mathrm{where}\;d_{i}\;=\;X_{i,\;bio}-\;X_{i,\;ctrl}$$

The threshold for statistical significance was set at *p* < 0.05, and all results are presented as mean ± standard error (mean ± SE).

## 3. Results

### 3.1. Analysis of Cerebral Blood Flow Dynamics in the DLPFC

**Figure 3:**
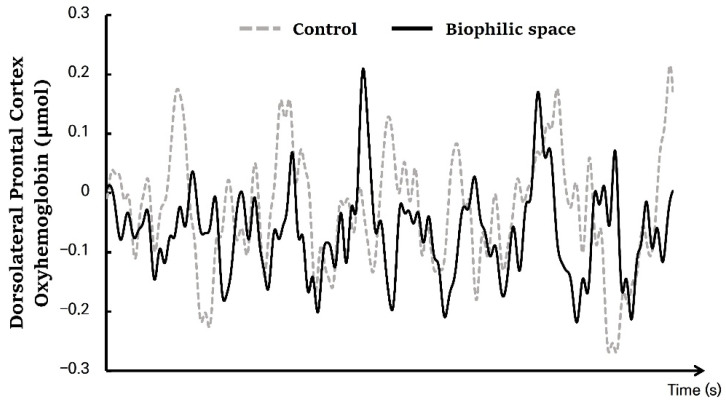
Temporal changes in oxyhemoglobin (oxy-Hb) concentrations in the dorsolateral prefrontal cortex (DLPFC) under the biophilic and control conditions. N = 21.

Real-time monitoring of the DLPFC revealed highly dynamic fluctuations in cerebral blood flow characterized by second-to-second rises and falls. This trend reflects the fact that prefrontal hemodynamic activity exhibits continuous fluctuations, even in the absence of specific task engagement (Figure 3). Observations of blood flow in the DLPFC across two different spatial conditions showed a general decrease relative to baseline, which can be interpreted as a natural decline associated with resting in a seated position. More importantly, this tendency is likely to be affected by the physical environment in the two different indoor spaces. To identify consistent patterns in continuous hemodynamic fluctuations, we segmented the data into fixed time intervals for analysis.

**Figure 4:**
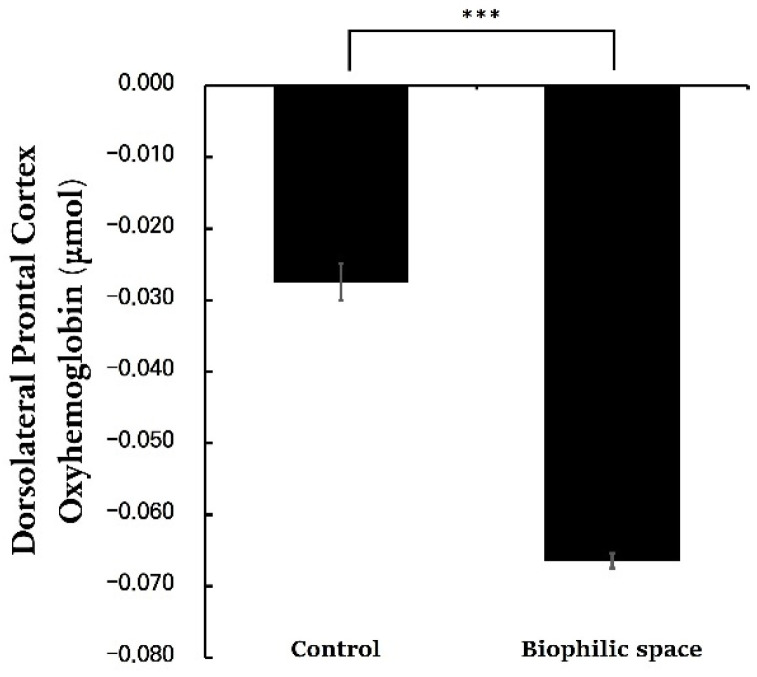
Mean oxyhemoglobin (oxy-Hb) concentrations in the dorsolateral prefrontal cortex (DLPFC) during the 10-min rest period under the control and biophilic conditions. N = 21; *** *p* < 0.001; Paired *t*-test.

When comparing blood flow levels at two-minute intervals between the control and biophilic conditions, the initial values were nearly identical; however, as time progressed, the experimental condition demonstrated a steeper decline (Table 2). Also, the comparison of 10-min averages revealed that the experimental group exhibited substantially lower mean hemodynamic activity in the DLPFC relative to the control group (Control, −0.030 ± 0.001 μmol/L; biophilic space, −0.066 ± 0.001 μmol/L, *p* < 0.001; Figure 4). Given that all environmental factors other than interior design were held constant, this difference can reasonably be attributed to the visual perception of spatial design.

### 3.2. Analysis of Psychological Restorativeness

Four standardized questionnaires were employed to assess participants’ psychological responses to two spatial settings with differing design attributes. The results across all measures exhibited generally consistent and statistically significant patterns. Clear differences were observed in the psychological responses between the control and biophilic spaces across the two conditions. In the biophilic space, scores on positive indicators were substantially higher, whereas scores on negative indicators were significantly lower.

**Figure 5:**
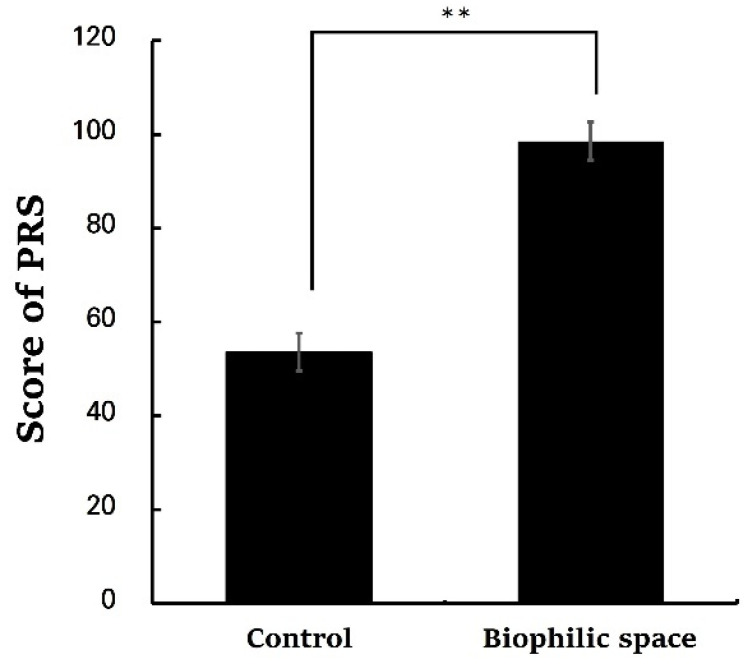
Total Perceived Restorativeness Scale (PRS) scores under the control and biophilic conditions. N = 21; ** *p* < 0.01; Wilcoxon signed-rank test.

**Figure 6:**
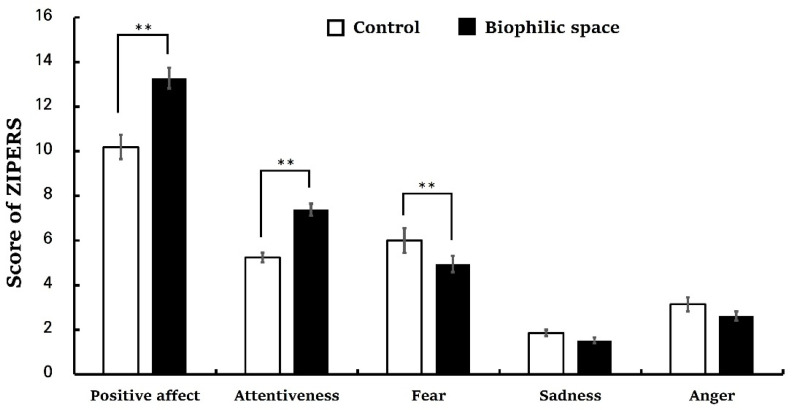
Subscale scores of the Zuckerman Inventory of Personal Reactions Scale (ZIPERS) under the control and biophilic conditions. N = 21; ** *p* < 0.01; Wilcoxon signed-rank test.

**Figure 7:**
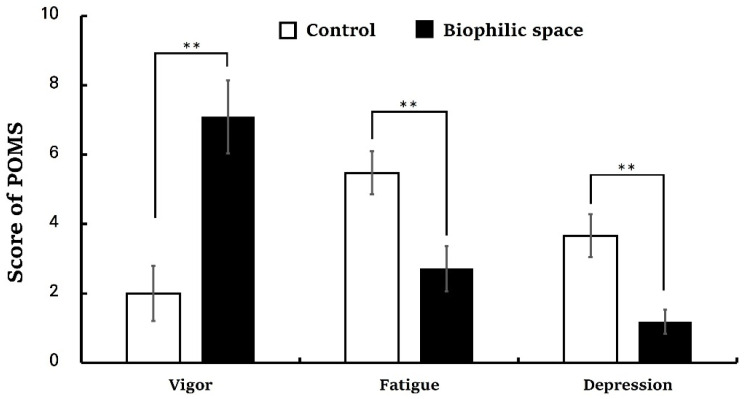
Profile of Mood States (POMS) scores under the control and biophilic conditions. N = 21; ** *p* < 0.01; Wilcoxon signed-rank test.

**Figure 8:**
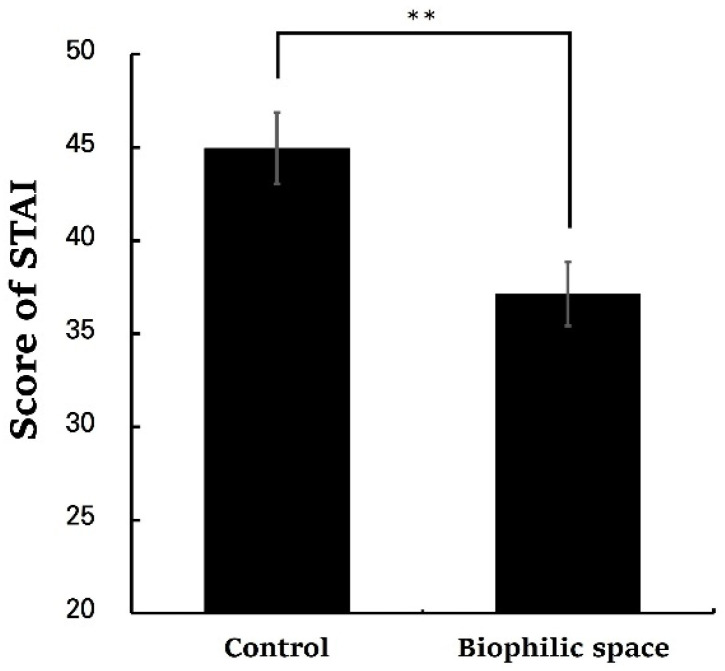
Total State–Trait Anxiety Inventory (STAI) scores under the control and biophilic conditions. N = 21; ** *p* < 0.01; Wilcoxon signed-rank test.

The analysis of the PRS revealed significantly higher total scores in the biophilic space condition compared to the control (control, 53.62 ± 3.98; biophilic space, 98.52 ± 4.07, *p* < 0.01; Figure 5). Subscales of PRS also demonstrated consistent trends: being away (control, 6.62 ± 0.96; biophilic space, 12.24 ± 0.80, *p* < 0.01), fascination (control, 9.48 ± 1.76; biophilic space, 27.38 ± 1.61, *p* < 0.01), coherence (control, 25.05 ± 1.05; biophilic space, 29.81 ± 1.10, *p* < 0.01), and compatibility (control, 12.48 ± 1.89; biophilic space, 29.10 ± 1.88, *p* < 0.01; Table 3). Fascination, in particular, exhibited a pronounced difference in the average values between the two. These results were consistent with the outcomes of the ZIPERS analysis (Figure 6), which indicated that mean scores for positive affect (control, 10.19 ± 0.54; biophilic space, 13.29 ± 0.47, *p* < 0.01) and attentiveness (control, 5.24 ± 0.22; biophilic space, 7.38 ± 0.27, *p* < 0.01) were significantly higher in the experimental condition than in the control condition. Conversely, the subscale of fear was elevated in the control group as compared to the experimental group (Control, 6.00 ± 0.55; biophilic space, 4.95 ± 0.37, *p* < 0.01). The results from the POMS indicated a parallel tendency (Figure 7): vigor (control, 2.00 ± 0.79; biophilic space, 7.10 ± 1.06, *p* < 0.01), representing a positive mood state, was substantially higher in the biophilic space, whereas the subscales of fatigue (control, 5.48 ± 0.62; biophilic space, 2.71 ± 0.65, *p* < 0.01) and depression (control, 3.67 ± 0.62; biophilic space, 1.19 ± 0.35, *p* < 0.01) showed significantly lower than in the control. Furthermore, analysis of the STAI confirmed that participants’ anxiety scores were significantly decreased in the biophilic space condition in comparison with the control (control, 44.95 ± 1.91; biophilic space, 37.14 ± 1.72, *p* < 0.01; Figure 8).

## 4. Discussion

In this study, we assessed the neuropsychological effects of two different indoor spaces and examined the impact of biophilic design on psychological restorativeness. The findings of this study not only demonstrated the direction and intensity of the psychological impacts with relative clarity but also provided scientific evidence necessary for exploring the mechanisms through which health-related effects are achieved under specific environments. The participant group in our study consisted of nurses working in a large hospital who are particularly susceptible to the influence of the physical environment in the workplace due to the extensive amount of time they spend indoors. Even with a short environmental exposure of 10 min, distinct differences in physiological and psychological responses between the two settings were clearly observed in this study.

The findings demonstrated that exposure to the biophilic condition significantly reduced oxyhemoglobin (oxy-Hb) concentrations in the DLPFC compared with the control condition. The DLPFC serves as a critical hub for executive functions, such as decision-making, attentional regulation, and emotional integration [40]. Activation of the DLPFC is often elevated in response to strong negative emotions or an increased emotional load [35]. It also mediates top-down regulation within the prefrontal–amygdala network, suppressing negative affect while enhancing positive emotion. The observed reduction in DLPFC activation in this study reflects a shift toward functional efficiency, indicating relief from cognitive–emotional overload [58].

The DLPFC plays an important role in modulating emotional valence [41] and connectivity within emotion regulation networks [39,42]. Based on fMRI studies, visual natural stimuli are transmitted via the visual and associative cortices to the PFC, where they stabilize emotional circuitry [24,25]. Earlier fMRI research has provided evidence supporting the validity of this consideration. Exposure to urbanized landscapes elevates amygdala activation, indicating that artificial environments can increase sensitivity to stress-related visual stimuli [23]. In contrast, exposure to natural environments significantly reduces amygdala activity, supporting the idea that nature mitigates the neurophysiological stress responses induced by urban settings [25].

Particularly, nurses are frequently exposed to high workloads, time pressure, and repeated encounters with patient suffering, which cumulatively lead to chronic occupational stress and emotional exhaustion. These conditions contribute to compassion fatigue and burnout, as consistently reported among emergency and surgical nurses [59,60,61]. A recent large-scale survey of over 70,000 U.S. nurses further revealed that approximately 26% of those who left the healthcare workforce between 2018 and 2021 cited emotional fatigue as the primary reason for resignation [62]. Collectively, these findings highlight that nurses working in highly artificial clinical environments are exposed to constant cognitive and emotional overload, suggesting that their physiological stress responses are closely intertwined with environmental factors. Consistent with this evidence, the nurses who participated in this study often face persistent cognitive and emotional overload in occupational settings—such as surgical units and other artificialized environments—which can result in excessive prefrontal hyperactivation that undermines performance and well-being [15]. Therefore, reduced prefrontal hemodynamic responses under biophilic exposure may be related to the functional optimization of neural processes because decreased DLPFC activity suggests neurochemical rebalancing—through neurotransmitters such as norepinephrine, dopamine, and acetylcholine—that facilitates cortical stabilization. Temporal variations in hemodynamic patterns may also represent adaptive neuroplastic processes by which the brain transitions into more efficient states.

The psychological data obtained in this study align with this perspective, demonstrating that the indoor biophilic space significantly improved psychological restorativeness. Exposure to such space significantly reduced negative emotional states, including tension, fatigue, depression, fear, and anxiety, while promoting positive affective dimensions, such as attentiveness and vitality. These findings are consistent with many previous studies that revealed that nature-based interventions positively influenced mental health and cognitive performance in occupational groups [18]. This trend has been explained by the Attention Restoration Theory, which posits that natural environments mitigate attentional fatigue and foster cognitive recovery [63].

Recent neuropsychological and environmental psychology studies have shown that natural environments reduce cognitive load and promote attentional restoration [26,58,64]. These findings suggest that biophilic design extends beyond its aesthetic role to function as a restorative environment that promotes neural efficiency and emotional resilience. The rapid onset of these benefits highlights the potential for integrating biophilic elements into everyday indoor environments for psychological restoration. Of particular significance is the observation that pronounced psychological benefits were evident despite brief 10-min exposure [65]. This suggests that even relatively short encounters with naturalized environments can exert measurable effects on affective states and cognitive functioning.

This study holds significance as it empirically verified the effects of biophilic design within real hospital environments. By simultaneously analyzing neurophysiological indicators (fNIRS-based hemodynamic changes) and psychological responses (environmental psychology measures), it provided a multidisciplinary understanding of how physical environmental stimuli influence prefrontal hemodynamics and psychological restoration. Conducting the experiment in an actual clinical setting with nurses—a high-stress occupational group—further strengthened the ecological validity of the findings and provided objective neurophysiological evidence for the restorative implications of biophilic design in healthcare environments.

Nevertheless, several limitations should be acknowledged. First, the relatively small sample size and short-term exposure period (10 min) restrict the generalizability of the findings and the assessment of long-term biophilic effects. Future studies should employ larger samples and repeated or prolonged exposure designs to investigate cumulative and sustained outcomes. Second, the possibility of self-selection bias cannot be fully excluded because this study involved voluntary participation. Subsequent studies should adopt random or stratified sampling methods to include more diverse occupational groups and age ranges, thereby improving generalizability. Third, the control condition was designed as a plain white wall, representing an extremely minimalized setting that may serve as an overly simplified contrast to the biophilic environment. While actual indoor environments typically include visual components such as furniture, color, and decorative elements, these factors were intentionally excluded to minimize design-related confounders. Given that white-wall conditions are commonly used in domestic and international studies as a neutral baseline, this approach allowed for the evaluation of pure environmental effects while maintaining the physical consistency of the space. Future research should explore variations in color schemes, material compositions, and furniture layouts to further elucidate how visual diversity affects restorative outcomes. Finally, this study conceptually focused on a single biophilic attribute—greenery exposure—to ensure experimental control and to represent the most fundamental and widely validated element of biophilic design. Although biophilic design encompasses multiple sensory-integrated attributes such as water, view, shelter, biodiversity, light, scent, and sound, plants serve as the most empirically supported and practically applicable medium in confined indoor environments. They directly embody the vitality and visual complexity of nature. Previous studies have demonstrated that responses to natural environments vary depending on setting type and vegetation density [66], suggesting that differences in landscape structure and sensory diversity may influence psychological recovery. Nonetheless, greenery provides a universally accessible and symbolically representative form of nature exposure, allowing for controlled and replicable investigation of biophilic mechanisms—particularly in clinical or laboratory contexts. Accordingly, this study adopted plant-based visual stimuli as a foundational step toward empirically validating the core mechanisms of biophilic design. Future studies should extend this work by comparing multiple design typologies—integrating elements such as water, flowers, shelter, and vistas—and examining how multisensory and compositional variations influence neurophysiological and emotional restoration.

Despite these limitations, the findings clearly demonstrate that biophilic design elements in artificial hospital environments can alleviate neurophysiological stress, enhance emotional recovery, and reduce occupational strain among healthcare professionals. For high-stress occupations such as nursing, introducing biophilic elements into accessible restorative spaces (e.g., 10 min breaks) represents a practical and compatible environmental strategy. This study therefore provides a valuable foundation for the standardized application of biophilic design in hospital architecture and interior planning, ultimately contributing to healthier and more restorative healthcare environments.

## 5. Conclusions

This study empirically demonstrated, via neurophysiological and psychological indicators, the effects of short-term exposure to indoor biophilic spaces on brain function and emotional recovery in artificial, enclosed environments. Specifically, the dorsolateral prefrontal cortex (DLPFC) exhibited a marked reduction in oxyhemoglobin concentration—over 50% lower than in the control condition—suggesting decreased cortical demand and enhanced neural efficiency under the biophilic setting. In parallel, multiple psychological indicators consistently reflected improved restorative outcomes, with the Perceived Restorativeness Scale (PRS) showing nearly double the score compared to the control condition, indicating a clear enhancement in perceived recovery and emotional stability. Together, these quantitative trends suggest that even brief exposure to natural elements may promote both cognitive relaxation and emotional stabilization.

These findings extend beyond immediate physiological responses, providing convergent evidence that indoor biophilic spaces may help mitigate health challenges associated with urbanization and the prevalence of highly artificialized environments. By integrating environmental psychology theories with neuroscientific evidence, this study contributes to advancing scholarly understanding of what constitutes a “healthy space.” Importantly, the implications of this study extend beyond particular occupational groups to a wide spectrum of work environments. Indoor biophilic spaces may function as essential restorative resources that promote recovery and well-being within urban contexts, suggesting their potential utility as practical strategies for healthier workplace designs.

On a broader scale, the findings may hold implications for urban planning and policymaking. Embedding restorative natural elements into a built environment could serve as a cost-effective, sustainable intervention to enhance public health, improve resilience to stress, and strengthen the overall livability of cities. Finally, by adopting an interdisciplinary approach bridging neuroscience, environmental psychology, and landscape architecture, this study provides a preliminary yet meaningful foundation for the incorporation of restorative landscapes into future urban design. In doing so, it suggests that biophilic interventions may improve individual health and contribute to the long-term sustainability and well-being of urban societies.

## Figures and Tables

**Table 1:** Plant species used in the biophilic space.

Scientific Name	Common Name
Rhapis excelsa	Lady Palms
Davallia mariesii	Squirrels-foot Fern
Dracaena sanderiana ‘Victoria’	Lucky Bamboo
Epipremnum aureum ‘Lime’	Lime Pothos
Trachelospermum asiaticum var. variegatum	Variegated Asiatic Jasmine
Hedera helix ’Sagittifolia’	English Ivy
Ardisia pusilla	Small Coralberry
Ardisia japonica	Marlberry
Chamaedorea elegans	Parlor palm
Hoya carnosa	Wax plant

**Table 2:** Mean oxyhemoglobin (oxy-Hb) concentrations in the dorsolateral prefrontal cortex (DLPFC) at 2-min intervals under the control and biophilic conditions. N = 21; *** *p* < 0.001; Paired-*t* test.

PFC Region	Sec.	Group	Mean	SE	*p*
DLPFC	0–120	Control	−0.028	0.003	<0.001
		Biophilic Space	−0.053	0.001	
	120–240	Control	−0.021	0.003	<0.001
		Biophilic Space	−0.087	0.002	
	240–360	Control	−0.032	0.002	<0.001
		Biophilic Space	−0.055	0.003	
	360–480	Control	−0.004	0.003	<0.001
		Biophilic Space	−0.047	0.003	
	480–600	Control	−0.064	0.004	<0.001
		Biophilic Space	− 0.091	0.002	

**Table 3:** Subscale scores of the Perceived Restorativeness Scale (PRS) under the control and biophilic conditions. N = 21; *** *p* < 0.01; Wilcoxon signed-rank test.

Score of PRS	Control		Biophilic Space		*p*
	Mean	SE	Mean	SE	
Being away	6.62	0.96	12.24	0.80	<0.01
Fascination	9.48	1.76	27.38	1.61	<0.01
Coherence	25.05	1.05	29.81	1.10	<0.01
Compatibility	12.48	1.89	29.10	1.88	<0.01

## Data Availability

The data that support the findings of this study are not publicly available due to privacy and ethical restrictions but are available from the corresponding author upon reasonable request.

## Abbreviations

The following abbreviations are used in this manuscript:

DLPFC	Dorsolateral Prefrontal Cortex
PFC	Prefrontal Cortex
OFC	Orbitofrontal Cortex
fMRI	Functional Magnetic Resonance Imaging
fNIRS	Functional Near-Infrared Spectroscopy
NIRS	Near-Infrared Spectroscopy
PRS	Perceived Restorativeness Scale
ZIPERS	Zuckerman Inventory of Personal Reaction Scale
POMS	Profile of Mood States
STAI	State-Trait Anxiety Inventory
SE	Standard Error
